# Supplementary material for: A mathematical-descriptor of tumor-mesoscopic-structure from computed-tomography images annotates prognostic- and molecular-phenotypes of epithelial ovarian cancer
Source: Nat Commun. 2019 Feb 15;10:764. doi: 10.1038/s41467-019-08718-9 (PMC6377605; doi:10.1038/s41467-019-08718-9)
Supplement: Supplementary file 1 — Supplementary Information [file 41467_2019_8718_MOESM1_ESM.pdf]

Supplementary Information

A mathematical-descriptor of tumor-mesoscopic-structure from computed-tomography images annotates prognostic- and molecular-phenotypes of epithelial ovarian cancer

Lu *et al.*

Supplementary Figures

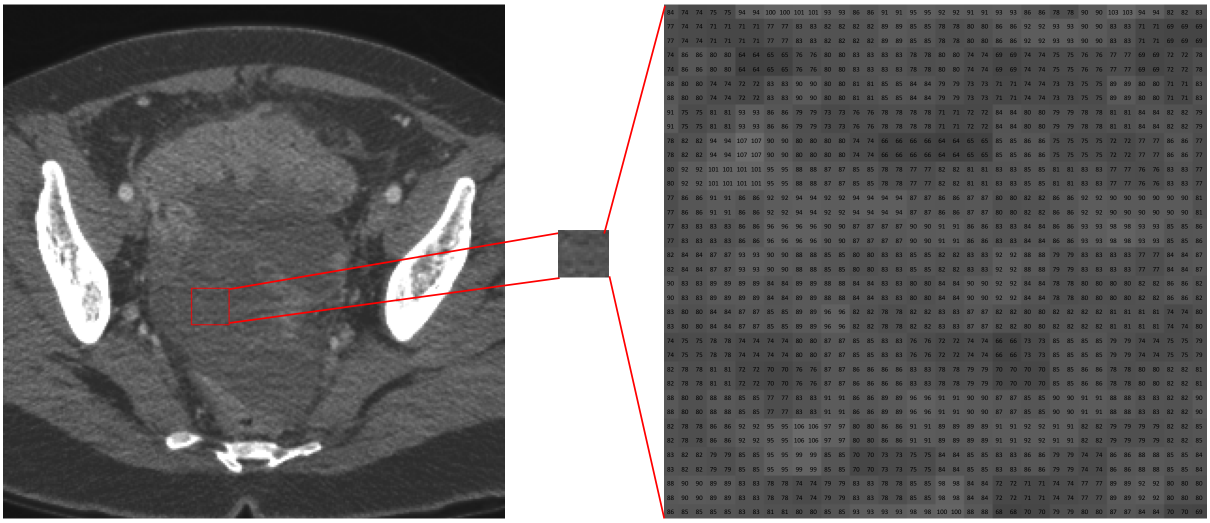

Supplementary Figure 1. An example of a septate cystic-solid ovarian tumour in the pelvis on an axial contrast enhanced CT scan. The histology was high grade serous ovarian carcinoma. On the right, a small sample through the cystic portion of the ovarian tumor was read by TexLab 2.0 and output as the statistical summary of the image. In our study, the entire tumour was segmented as a single 3-D mass.

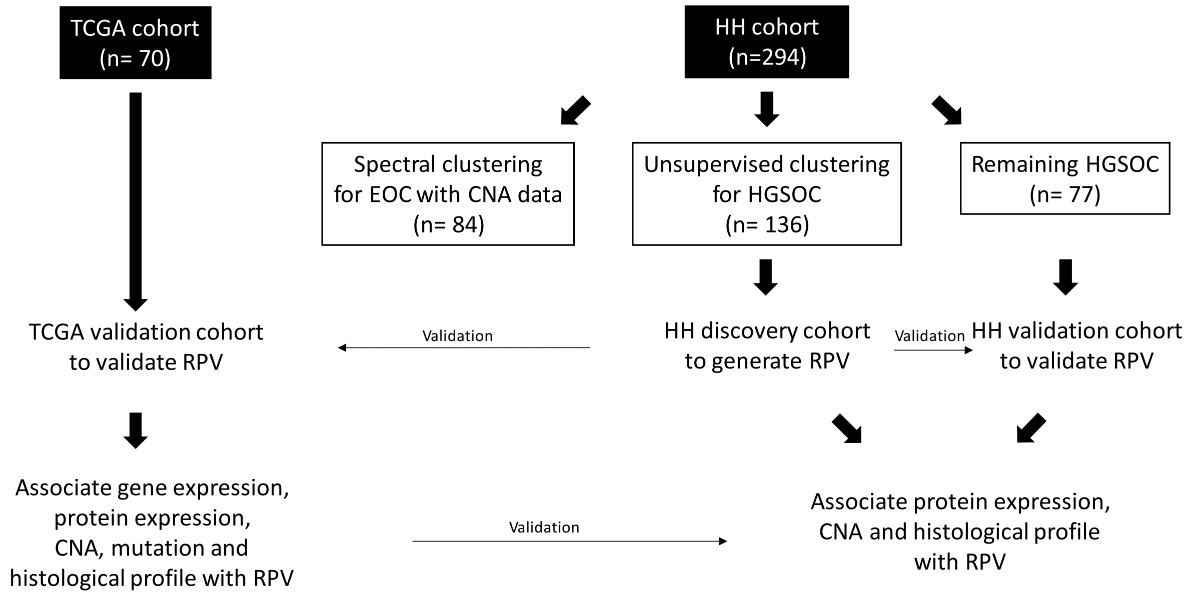

Supplementary Figure 2. Study workflow. In total, 294 Computerised Tomography (CT) images for ovarian cancer patients were collected retrospectively from Hammersmith hospital (HH cohort). The primary tumors from CT images were segmented by experienced radiologists and analyzed by TexLab 2.0 software to generate a radiomic profile for each patient. Fresh frozen tumor samples were available for a subset of cases and sent for indicated molecular profiling. In addition to the Hammersmith cohort, 70 cases from the TCGA ovarian cancer dataset with corresponding molecular profiles were used as a validation cohort.

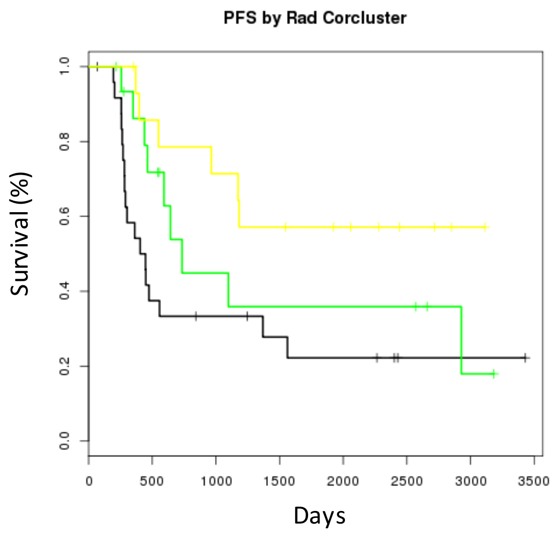

Supplementary Figure 3. Clinical association with radiomic similarity and hierarchical clusters. The association of radiomic similarity clusters and progression free survival. P= 0.0338, n= 56, log-rank test. Black, Group 1; Yellow: Group 2; Green: Group 3.

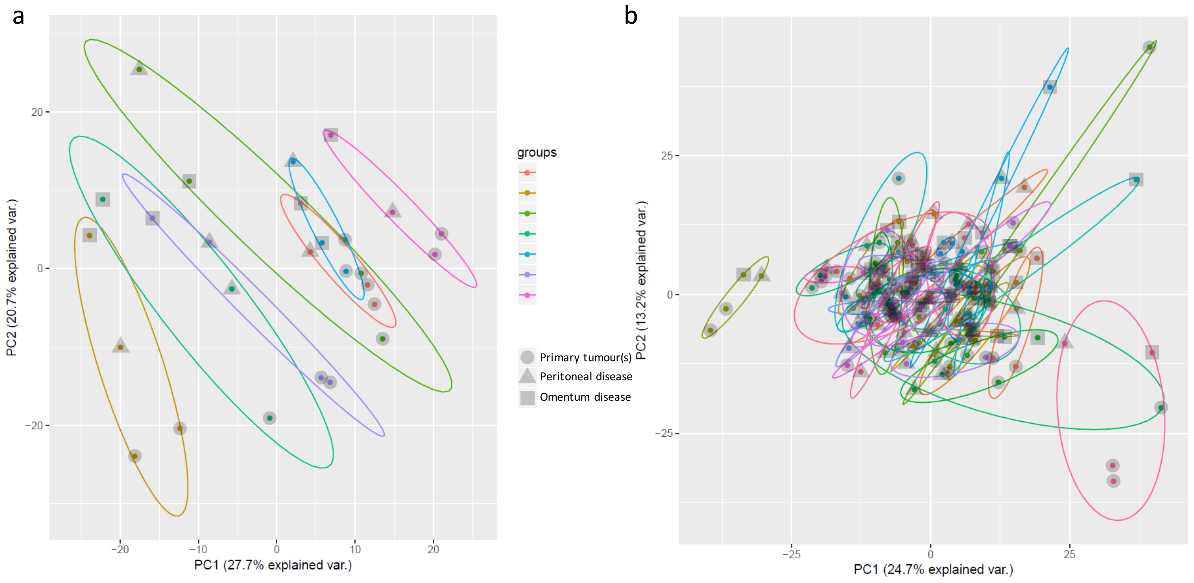

Supplementary Figure 4. Principal component analysis of radiomic profiles from primary tumors, disease in the omentum and peritoneum for (a) 7 cases and (b) 48 cases. Each color represents a HGSOC case and each shape represents a disease location.

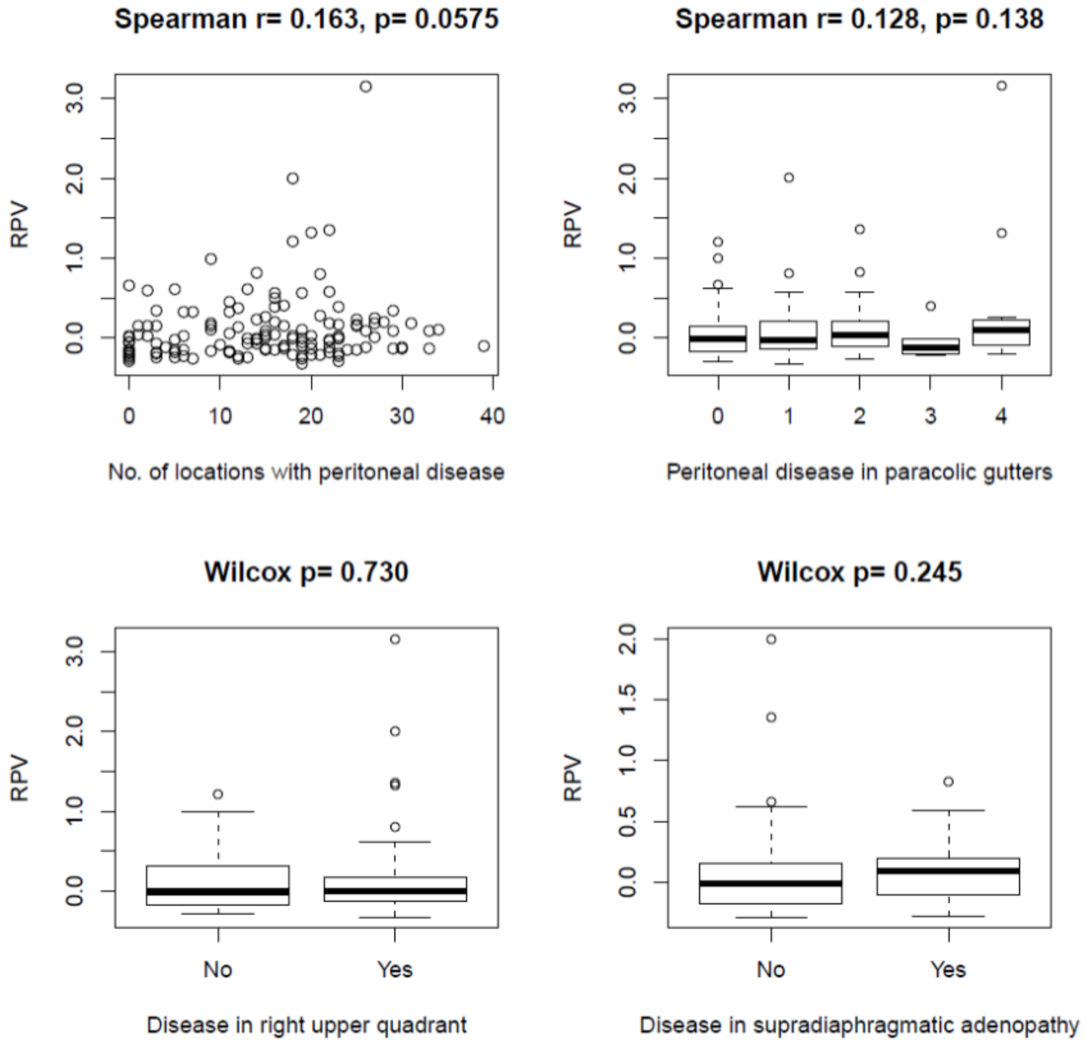

Supplementary Figure 5. Association between RPV and number of locations with peritoneal disease, peritoneal disease in paracolic gutters, peritoneal disease around liver/right upper quadrant or supradiaphragmatic adenopathy in the HH cohort. Box indicates median (thick line) and quartiles (thin line); whiskers represent 1.5x interquartile range; points are outliers.

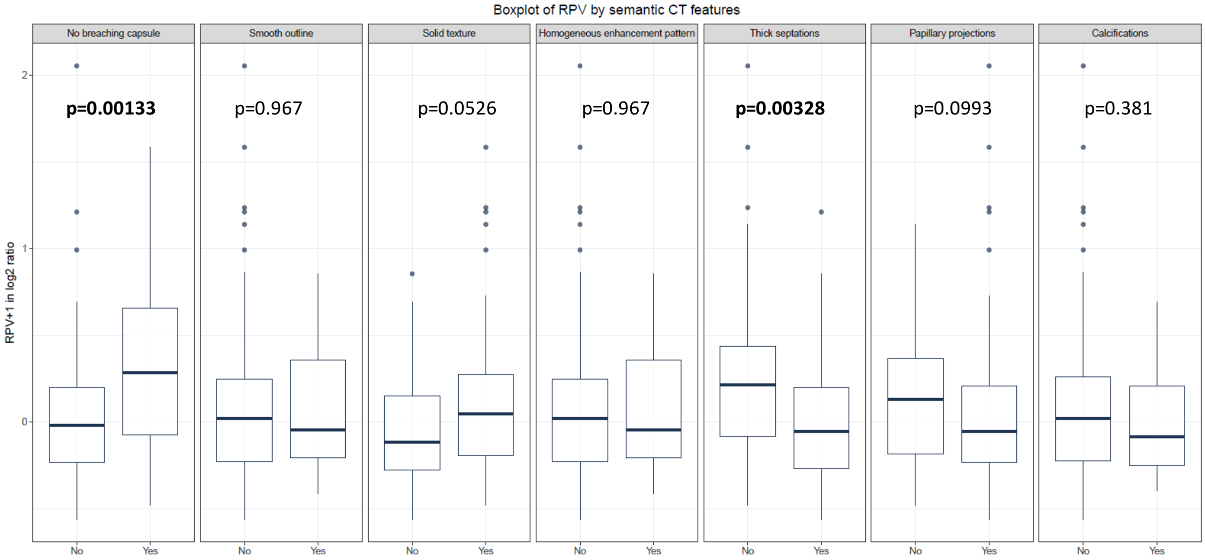

Supplementary Figure 6. Association between RPV and breaching capsule, smooth outline, solid texture, homogeneous enhancement pattern, presence of thick septations, papillary projections or calcifications in the primary tumors in the HH cohort. P-values are given by two-tailed Wilcoxon rank-sum test.

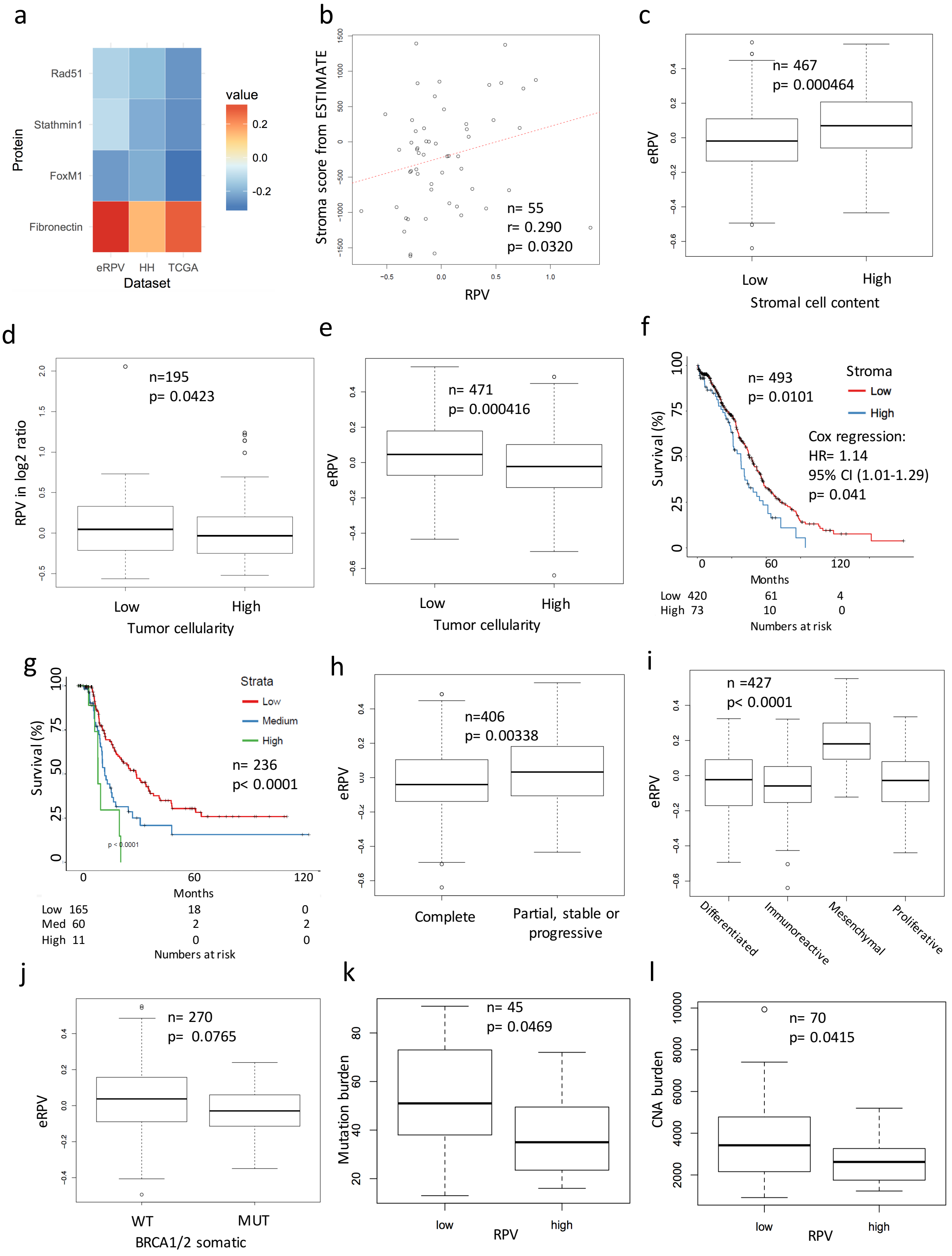

Supplementary Figure 7. Biological and clinical association with RPV. (a) Protein expression correlated with RPV in TCGA and HH datasets and with eRPV in additional TCGA dataset. (b) Scatter plot showing the correlation between RPV and stroma score from ESTIMATE. The Spearman correlation coefficient and p-value are indicated on the scatter plot. Association between eRPV and (c) stromal cell content and (e) tumor cell content in the additional TCGA dataset. P-value is given by two-tailed Wilcoxon rank-sum test. (d) RPV level association with tumor cellularity in HH cohort. The cut-off between low and high tumor cellularity is 30% of tumor cells present in the histological sections. P-value is given by two-tailed Wilcoxon rank-sum test. (f) Kaplan-Meier analysis of stromal

cell content and overall survival in TCGA dataset. High and low stromal cell content is defined at upper-quantile of stromal cell percentage. P-value is given by log-rank test. Result from continuous Cox regression analysis adjusted for stage and residual disease is indicated. (g) Kaplan-Meier analysis of RPV and PFS in the combined datasets. P-value is given by log-rank test. (h) Association between eRPV and initial chemotherapy response. P-value is given by two-tailed Wilcoxon rank-sum test. (i) The association between eRPV and 4 molecular subtypes in the additional TCGA dataset[14]. P-value is given by Kruskal-Wallis test. (j) eRPV level association with somatic BRCA1/2 mutation in TCGA dataset. P-value is indicated from two-tailed Wilcoxon rank-sum test. Association between (k) mutation burden or (l) CNA burden with RPV in the TCGA dataset. Mutation burden from Exome sequencing is defined by the number of genes mutated from Exome sequencing. CNA burden is defined by total number of genes with amplification or deletion per case. P-values are given by two-tailed t-test.

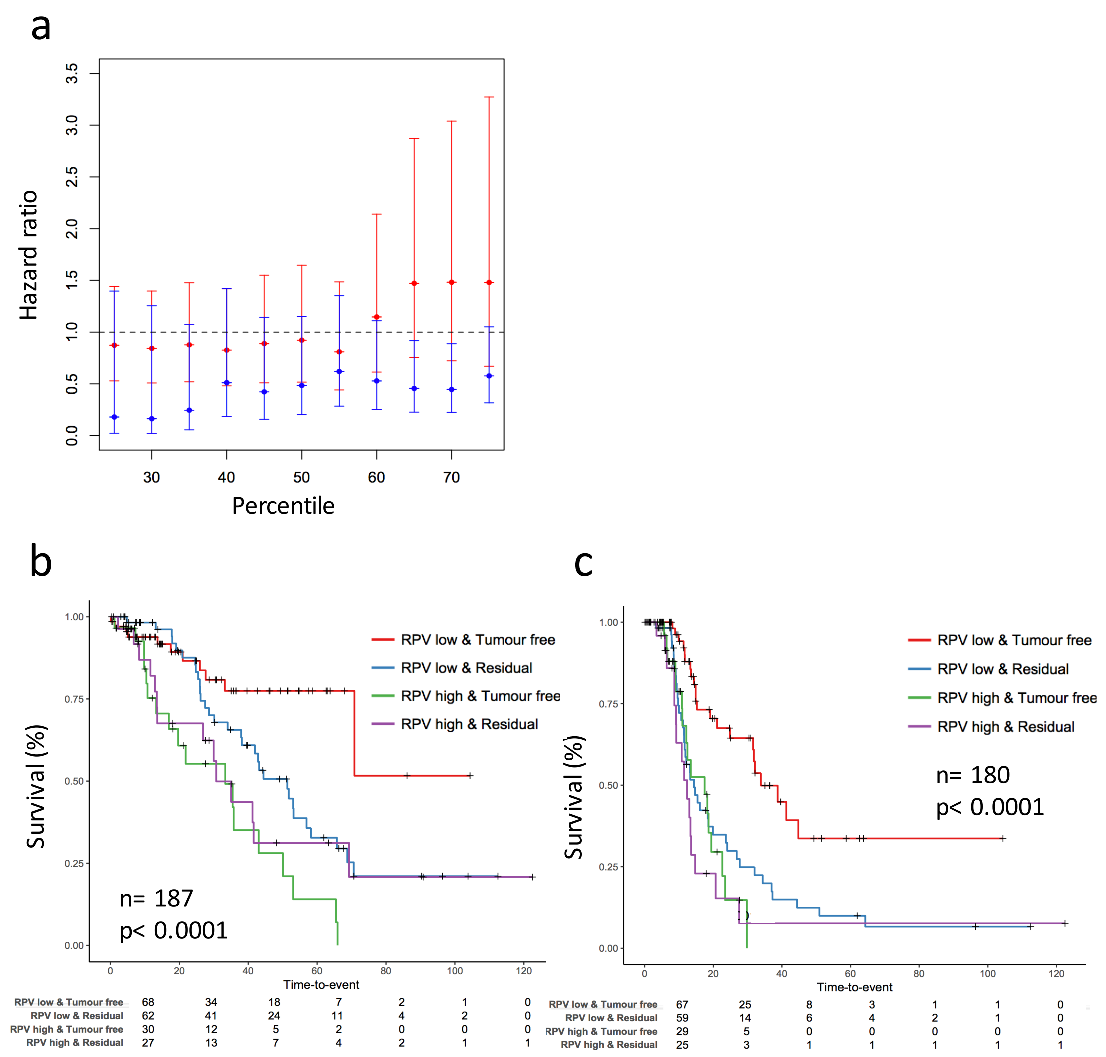

Supplementary Figure 8. RPV as a potential predictive marker for surgical response. (a) Hazard ratio of post-operative tumor free status using overall survival as outcome in RPV-high (red) and RPV-low (blue) groups defined by a range of cut-off values (x-axis) in the combined dataset. 95% CI for each hazard ratio is indicated as error bars. The HH and TCGA cohorts were combined due to the limited number of post-operative tumor-free patients. Stage III-IV patients are included. Kaplan-Meier analysis of (b) overall survival and (c) progression free survival in patients defined by RPV status and post-operative residual disease. RPV at 70% percentile is used to define RPV-high and RPV-low since HR from the two groups separate mostly at this cut-off. P-value is given by log-rank test. The number of cases at risk is indicated below the plots.

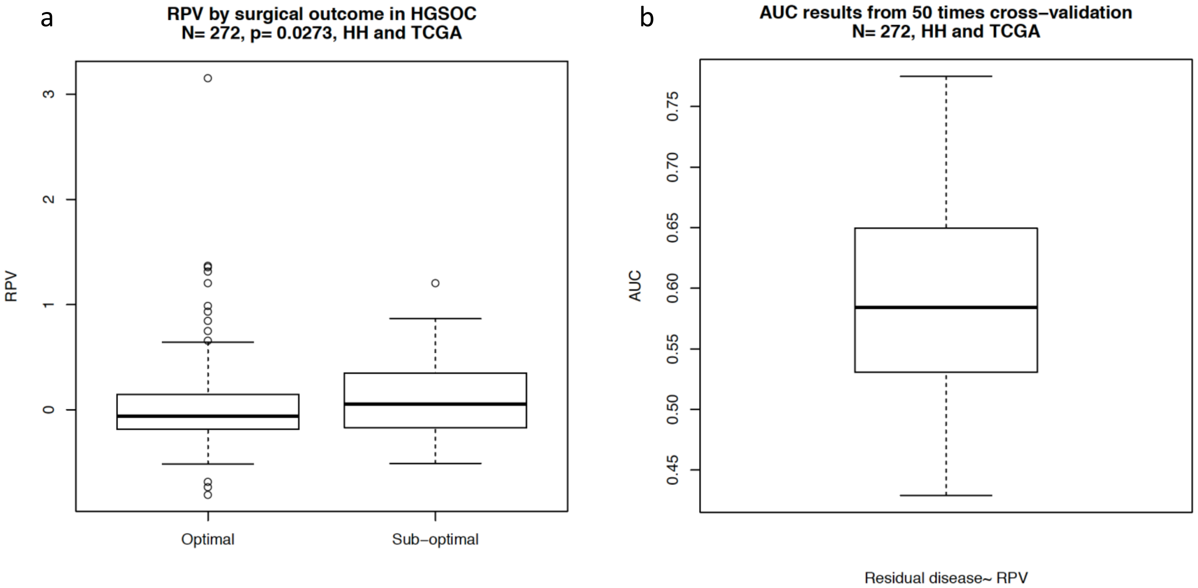

Supplementary Figure 9. Association between RPV and sub-optimal resection. (a) High RPV was associated with sub-optimal resection in the HH and TCGA cohort. P-value is given by two-tailed Wilcoxon rank-sum test. (b) Summary of area under curve (AUC) in the validation sets after random splitting the HH and TCGA cohort with ratios of 7 (training using logistic regression) to 3 (validation) for 50 times. The average AUC is 0.58.

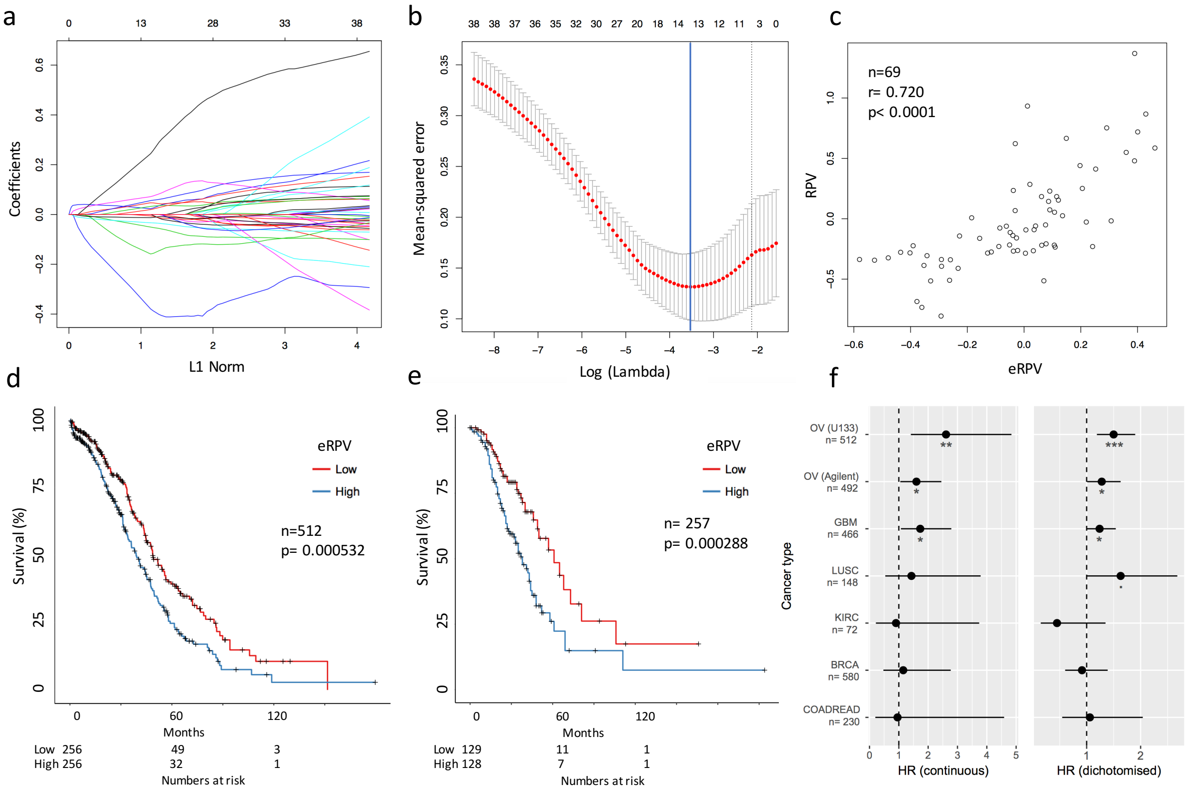

Supplementary Figure 10. Construction of eRPV based on gene expression profile in the TCGA datasets and Tothill dataset. Selection of gene expression features to recapitulate RPV using LASSO is summarised in (a) and (b). (a) Feature coefficients were plotted against shrinkage parameter (Lambda) after performing linear regression between gene expression with RPV using LASSO in the TCGA validation dataset. (b) Selection of Lambda minimum after 10-fold cross-validation. The number of gene expression features are on the top x-axis. The Lambda minimum which resulted in the least error after cross-validation of regression between weighted expression level of 13 genes and RPV is highlighted in blue. (c) Correlation between eRPV and RPV in the TCGA validation dataset. Pearson correlation coefficient and p-value is indicated. eRPV association with OS in (d) the additional TCGA cohort without publicly available CT scans and (e) Tothill cohort. P-values are given by log-rank test. (f) Forest plot showing hazard ratio of eRPV with OS in ovarian cancer (eRPV generated from two microarray platforms) and five other cancer types in TCGA dataset. Continuous HR between eRPV and OS is given on the left and median dichotomised HR is on the right. *** p< 0.001, ** p< 0.01, * p< 0.05, • p< 0.1.

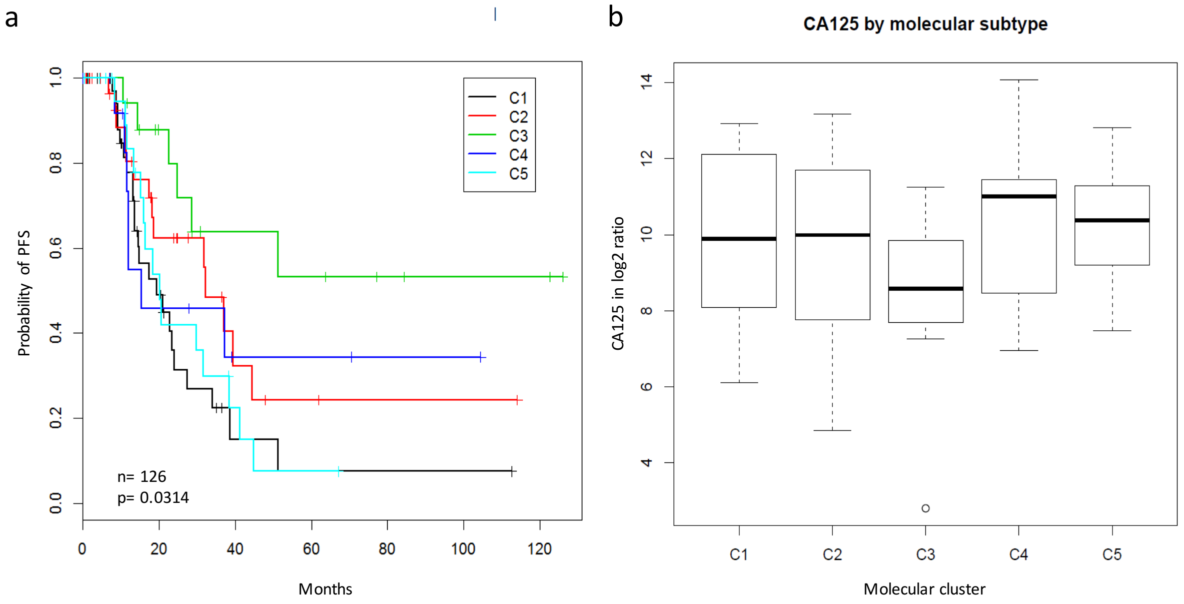

Supplementary Figure 11. (a) Kaplan-Meier analysis of molecular subtypes and progression free survival in the HH dataset. P-value is given by log-rank test. (b) Association between CA125 level and molecular subtypes.

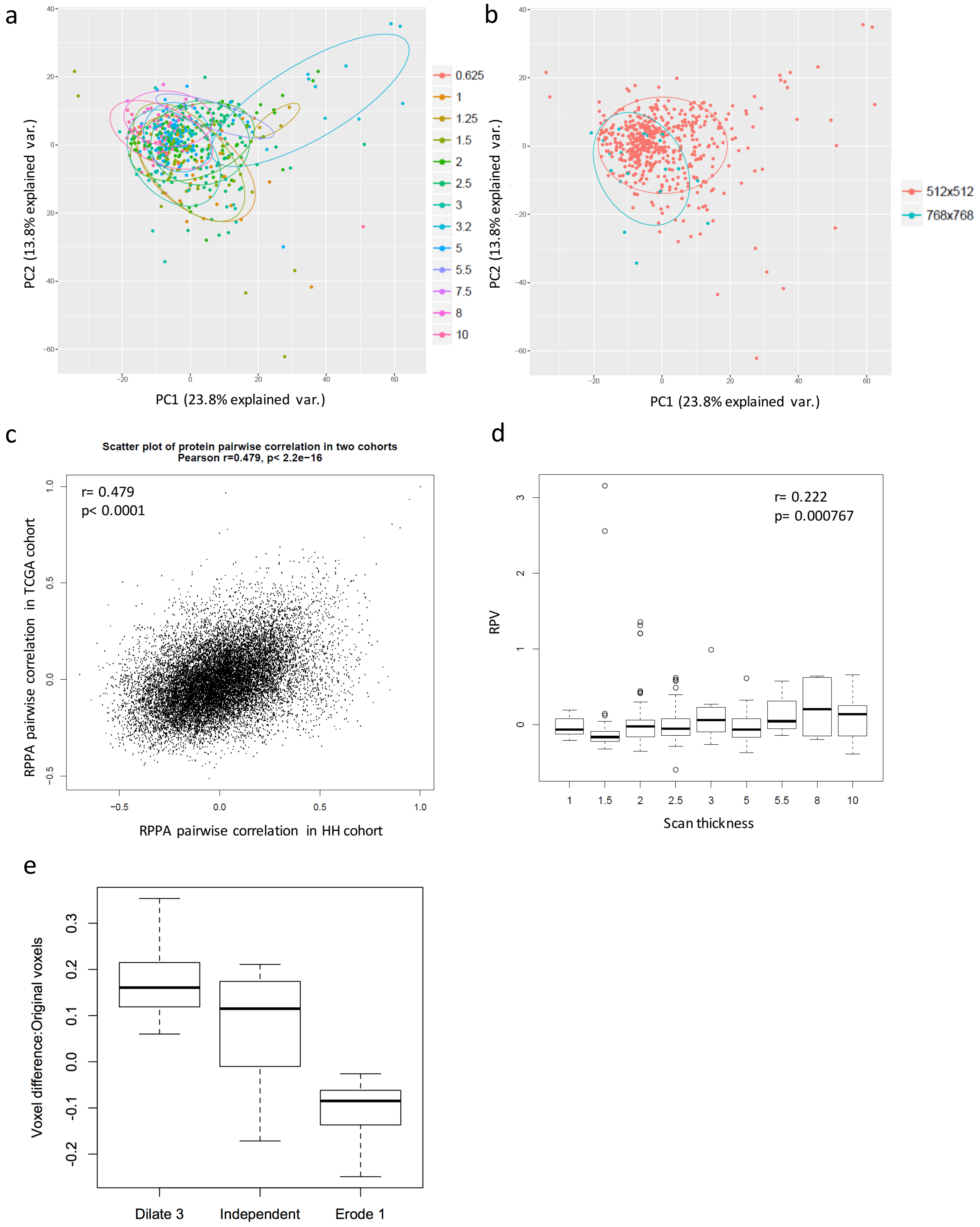

Supplementary Figure 12. Principal component analysis of radiomic profile annotated by (a) CT scan thickness and (b) matrix. (c) Feature-wise correlation from RPPA in HH cohort (x-axis) compared with TCGA cohort (y-axis). The Pearson correlation coefficient and p-value are indicated. (d) Correlation between RPV and scan thickness in HH cohort. Spearman correlation coefficient and p-value are indicated. (e) The range of difference in 21 independent segmentations compared to dilation by 3 pixels and erosion by 1 pixel. Y-axis indicates the ratio between difference in number of voxels and number of original voxels.

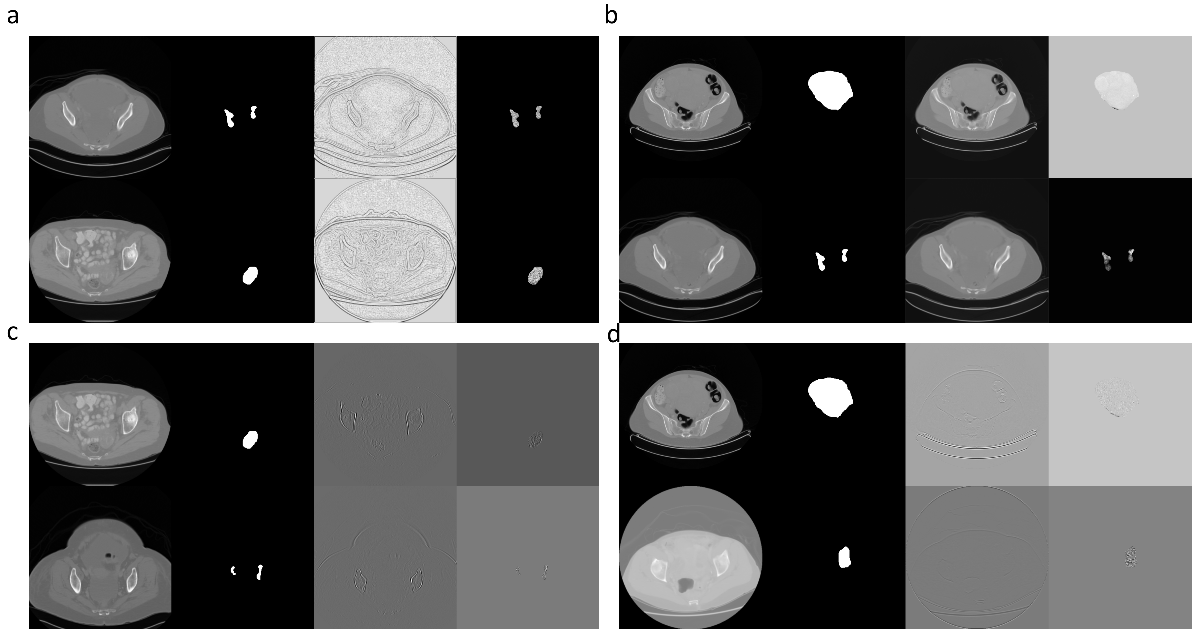

Supplementary Figure 13. Intermediate filtered CT images that are used to compute the final 4 radiomic features included in the construction of RPV. CT images filtered to visualise (a) FD_max_25HUgl: fractal dimension transformed image; the feature is then taken as the max value within the tumor mask. (b) GLRLM_SRLGLE_LLL_25HUgl: wavelet filtered image, which is then used to compute the short run grey length in all 26 directions in the 3D image, taking the average of all directions as final value. (c) FOS_Imedian_LHH: wavelet filtered image; the median value within the tumor mask is then taken as final value. And (d) NGTDM_Contra_HLL_25HUgl: wavelet filtered image, which is then used to compute the contrast within the tumor mask. The CT scan with minimum value (top panel) of the radiomic feature is compared with the CT scan with maximum value (lower panel) of the corresponding feature. The four images for each CT scan indicates (from left to right) image, mask, filtered image, masked fractal dimension image. Applying a wavelet filter on a CT image with a given set of filtering parameters can reveal textures with specific properties. For example, a high-pass wavelet filter reveals image areas with fine or rapid changing texture; on the other hand, a low-pass wavelet filter is the opposite of a high-pass one, and it "smooths/blurs" the image by averaging out the rapid changing texture.

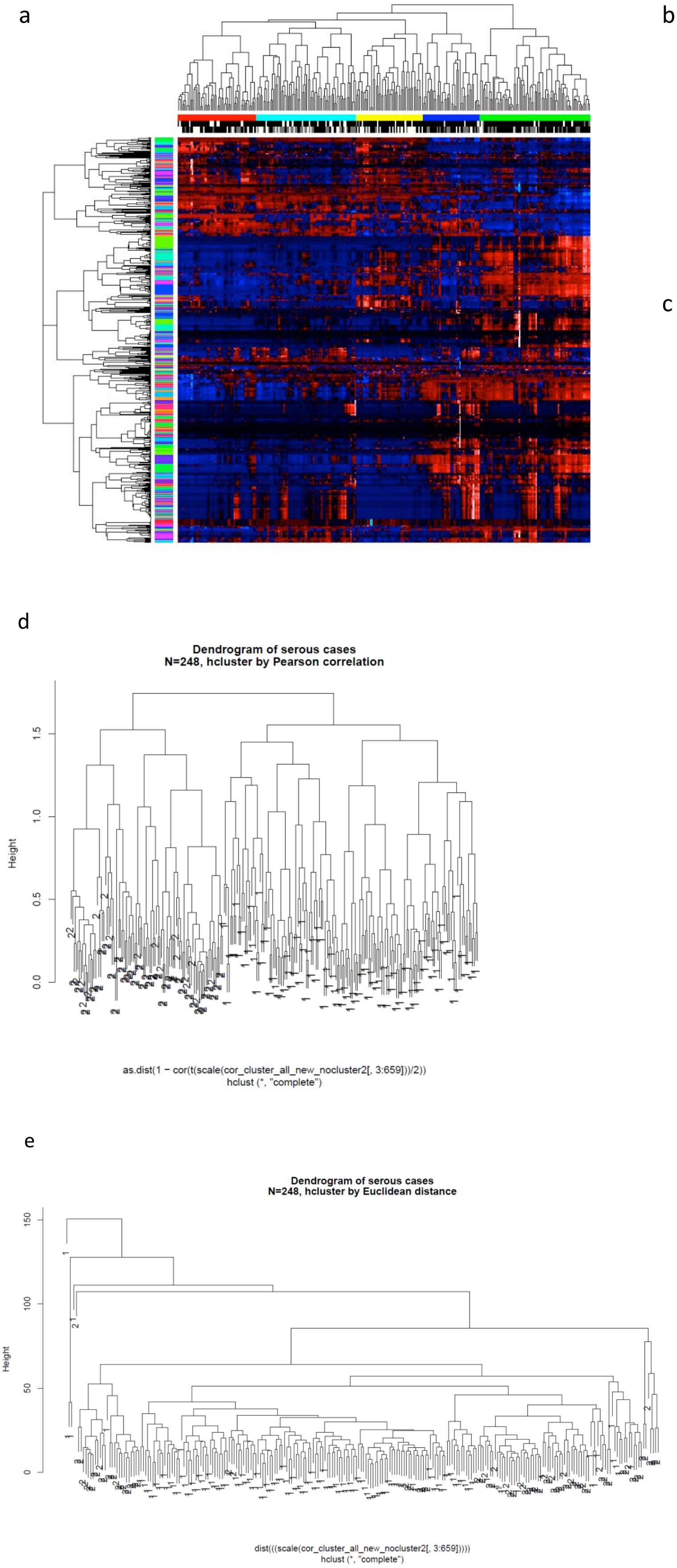

Supplementary Figure 14. Optimisation of hierarchical clustering using radiomic profile of HGSOC tumors. (a) Unsupervised hierarchical clustering using all HGSOC tumors (including bilateral tumors)

in the HH cohort. All tumors were split into five subgroups. (b) Kaplan-Meier analysis showing the association of the five subgroups with progression free survival. P-value is given by log-rank test. (c) Boxplot showing the scan thickness of tumours in the HH cohort by subgroups. Subgroup 2 showed significantly different scan thickness and survival compared to other subgroups. P-value is given by Kruskal-Wallis test. After removing subgroup 2, dendrograms were plotted using sample-wise distance measured by (d) Pearson correlation and (e) Euclidean distance.

Supplementary Tables

Supplementary Table 1. Clinical characteristics of patients in the three datasets.

		HH cohort all (N=294)	HH discovery cohort (N=136)	HH validation cohort (N=77)	TCGA validation cohort (N=70)
Age at diagnosis	Median	62	61	66	59
	Range	19-91	32-91	31-84	40-81
Stage	I-II^	53	20	5	13
	III-IV	223	115	71	57
	Unknown	18	1	1	0
Grade	Low	16	0	0	0
	High	254	136	77	69
	Unknown	24	0	0	1
Histology	Serous	231	136	77	70
	Non-serous	58	0	0	0
	Unknown	5	0	0	0
Neo-adjuvant chemotherapy	No	240	136	37	70
	Yes	43	0	38*	0
	Unknown	11	0	2	0
Residual disease	<10mm	205	97	57	50
	>10mm^	59	36	14	15
	Unknown	30	3	6	5
Relapsed	No	131	59	34	39
	Yes	132	77	29	25
	Unknown	31	0	14	6
Progression free survival (Months)	Median	23.1	21.9	16.4	19.1
	95% CI	18.3-33.9	16.1-31.6	13.5-44.4	12.0-27.7
Deceased	No	172	79	45	51
	Yes	115	57	32	19
	Unknown	7	0	0	0
Overall survival (Months)	Median	53.2	53.2	34.8	56.9
	95% CI	44.4-69.3	43.1-69.3	30.4-57.2	37.9-70.6

*Only CT scans obtained prior to neo-adjuvant chemotherapy were used for the radiomic analysis.
^Note that only a small proportion HGSOC patients with early stage disease or sub-optimal resection were included.

Supplementary Table 2. Radiomic features and their weightings contributing to RPV.

Features	Coefficient
FD_max_25HUgl	-0.0876
GLRLM_SRLGLE_LLL_25HUgl	0.0869
NGTDM_Contra_HLL_25HUgl	0.165
FOS_Imedian_LHH	0.250

Supplementary Table 3. The range of RPV-stratified risk groups.

Groups	RPV range
Low risk	Minimum- 0.0950
Medium risk	0.0950- 0.658
High risk	0.658- Maximum

Supplementary Table 4. Univariate and multivariable Cox regression analysis for overall survival in the combined HH and TCGA cohort.

		Univariate		Multivariable	
HH discovery + HH validation + TCGA validation (n= 82)	Variables	HR (95% CI)	p-value	HR (95% CI)	p-value
	RPV	3.96 (2.07-7.57)	3.22×10^{-5}	4.19 (2.042-8.61)	9.44×10^{-5}
	Stage	1.64 (0.560-4.80)	0.367	2.37 (0.730-7.71)	0.151
	Residual disease	3.26×10^{-9} (0-Inf)	0.998	3.57×10^{-9} (0.00-Inf)	0.998
	Age	0.964 (0.915-1.02)	0.167	0.966 (0.914-1.02)	0.220
	Performance status (ECOG)*	0.924 (0.385-2.22)	0.859	1.50 (0.602-3.72)	0.386
	* Only cases with available performance status were included.				

Supplementary Table 5. Comparison between RPV and existing prognostic markers in HGSOC in univariate and multivariable Cox regression analysis.

	Dataset	Univariate	Multivariable
Molecular subtype	HH (PFS)	1.88 (1.04-3.41), p=0.0359	1.70 (0.924-3.13), p=0.0880
RPV	N=95	3.64 (1.59-8.33), p=0.00226	3.13 (1.38-7.13), p=0.00657
Molecular subtype	HH (OS)	1.20 (0.569-2.51), p=0.637	0.937 (0.423-2.08), p=0.872
RPV	N=95	4.43 (2.43-8.08), $p=1.22x10^{-6}$	4.49 (2.40-8.40), $p=2.57x10^{-6}$
Molecular subtype	Tothill (PFS)	2.13 (1.54-2.96), $p=5.09x10^{-6}$	1.44 (0.963-2.15), p=0.0765
eRPV	N=263	11.7 (4.33-31.4), $p=1.18x10^{-6}$	9.22 (2.38-35.8), p=0.00132
Molecular subtype	Tothill (OS)	1.89 (1.26-2.83), p=0.00205	1.28 (0.806-2.05), p=0.293
eRPV	N=263	13.7 (3.81-49.0), $p=6.06x10^{-5}$	13.1 (2.50-69.0), p=0.00236
CLOVAR	Tothill (OS)	2.56 (1.73-3.80), $p=2.75x10^{-6}$	2.35 (1.57-3.51), $p=2.92x10^{-5}$
eRPV	N=263	13.7 (3.81-49.0), $p=6.06x10^{-5}$	5.38 (1.46-19.7), p=0.0113
CA125	HH (OS)	1.24 (1.01-1.53), p=0.0376	1.27 (1.03-1.57), p=0.0272
RPV	N=121	4.08 (2.48-6.71), $p=3.37x10^{-8}$	3.81 (2.26-6.43), $p=5.04x10^{-7}$

Supplementary Table 6. Univariate and multivariable Cox regression analysis for overall survival in the HH cohort (including radiology features that have been previously reported as prognostic).

		Univariate		Multivariable	
HH cohort (n=97)	Variables	HR (95% CI)	p-value	HR (95% CI)	p-value
	RPV	2.56 (1.38-4.76)	0.00288	5.11 (1.26-20.8)	0.0225
	No. of locations with peritoneal disease	1.05 (1.02-1.08)	0.000649	0.989 (0.933-1.05)	0.707
	Peritoneal disease in paracolic gutters	1.49 (1.19-1.86)	0.000527	1.66 (1.13-2.44)	0.00936
	Peritoneal disease around liver/right upper quadrant	1.98 (1.14-3.47)	0.0163	1.80 (0.710-4.54)	0.216
	Supradiaphragmatic adenopathy	3.66 (1.89-7.08)	0.000116	2.50 (1.135-5.51)	0.0230
	Stage	1.63 (1.06-2.49)	0.025	1.02 (0.562-1.85)	0.952
	Residual disease	1.15 (0.621-2.12)	0.658	0.700 (0.309-1.59)	0.394
	Age	0.999 (0.977-1.02)	0.951	1.01 (0.982-1.05)	0.413

Supplementary Table 7. Univariate and multivariable Cox regression analysis for overall survival in the HH cohort (including semantic radiology features).

		Univariate		Multivariable	
HH cohort (n=124)	Variables	HR (95% CI)	p-value	HR (95% CI)	p-value
	RPV	2.85 (1.77-4.57)	1.48×10^{-5}	3.25 (1.81-5.82)	7.47×10^{-5}
	Breaching Capsule	1.08 (0.576-2.03)	0.81	0.678 (0.331-1.39)	0.288
	Thick septations	0.727 (0.403-1.31)	0.291	0.838 (0.427-1.64)	0.606
	Stage	1.63 (1.06-2.48)	0.025	1.46 (0.936-2.28)	0.0949
	Residual disease	1.15 (0.621-2.12)	0.658	1.09 (0.571-2.07)	0.798
	Age	0.999 (0.977-1.02)	0.951	0.999 (0.973-1.03)	0.936

Supplementary Table 8. Potential therapeutic targets in the RPV-stratified patient groups.

RPV status	Therapeutic target	Evidence	Clinical status for HGSOC
High	CCND1	Over-expression	Under clinical development e.g. NCT02657928
	Angiogenesis	PECAM1 over-expression	Approved*
	mTOR	Activated pathways	Under clinical development e.g. ISRCTN16426935
Low	PARP	PARP2 over-expression; Activated pathways	Approved
	Immune system	Tumor mutational burden	Under clinical development e.g. NCT02766582
	Proteasome	Over-expression; Activated pathways	Under clinical development e.g. NCT02211755
*Bevacizumab. Only 3 cases were treated with Bevacizumab in the current patient cohort.			

Supplementary Note 1

Clustering of bilateral tumors

Approximately 30% of HGSOC patients present with tumors in both ovaries and previous genetic studies of bilateral tumors reveal close similarity[1]. To understand the radiomic characteristics of bilateral tumors, we included both primary tumors from patients with bilateral disease (n = 90 patients) in the hierarchical clustering analysis. We found that 96% of bilateral tumors clustered into the same subgroup despite their frequent dissimilarities in shape and size (Fig. 1d). Consistently, principal component analysis of the radiomic profile from bilateral tumours of the same patient also grouped closely (Supplementary Figure 4). In contrast, radiomic profiles from the diseases in the omentum and peritoneum were not closely grouped with their primary tumors, and clustered in a more complex way compared to the two primary tumors.

This result confirmed that bilateral tumors from the same HGSOC patient are closely related radiologically, thus the radiomic profile from only one of the bilateral tumors was chosen for subsequent analysis.

RPV predicts survival in synergy with existing CT-based morphological approaches

Although there is no clinically-approved approach to predict overall survival for HGSOC patients based on CT scans, a few CT characteristics summarised by radiologists were linked to prognosis in HGSOC[2]. To compare the prognostic performance of RPV with the existing radiology-based features, we collected four features (Number of locations with peritoneal disease; Peritoneal disease in paracolic gutters; Peritoneal disease around liver/right upper quadrant; Supradiaphragmatic adenopathy) that were previously described to predict survival in HGSOC[2].

We firstly confirmed that all of these four radiology features associated with overall survival. In the multivariable Cox regression model, RPV was found associated with overall survival independent of

the four radiology features (Supplementary Table 6). Interestingly, two other radiology features (Peritoneal disease in paracolic gutters and Supradiaphragmatic adenopathy) remained significant as covariates together with RPV, suggesting that a radiomics-based approach could potentially be used in combination with conventional radiology features. Furthermore, we did not observe any significant correlation between RPV with any of the prognostically-relevant radiology features, which further confirms that they are independent of each other (Supplementary Figure 5).

In addition to the prognostically relevant radiology features, we also collected a set of radiology-based features including breaching capsule, smooth outline, solid texture, homogeneous enhancement pattern, presence of thick septations, papillary projections, and calcifications in the primary tumors (Supplementary Figure 6). We found that RPV was higher in tumors without breaching capsule and tumors without thick septations. However, these two radiology-based features did not interact with the prognostic value of RPV in the multivariable Cox regression model (Supplementary Table 7).

CT scan is currently the standard of care imaging modality for HGSOC patients and only a small proportion of patients will have PET, MRI or ultrasound, which makes CT the most attractive imaging modality for HGSOC.

To conclude, our results suggest that RPV does not derive from the CT-based morphological features, but could potentially synergise with these radiology-based approaches.

Potential application of RPV in the surgical setting

The presence of post-operative residual tumor is a poor prognostic factor in HGSOC, although a subset of patients will have a poor prognosis despite achieving optimal residual disease status [10]. After stratifying patients into RPV-high (n= 57) and RPV-low (n= 130) groups, we found that tumor-free status was significantly associated with increased OS (HR: 0.446, 95% CI 0.223-0.889; p= 0.0217)

and PFS (HR: 0.379, 95% CI 0.225-0.639; p= 0.000275) only in the RPV-low group, not in the RPV-high group (Supplementary Figure 8b-c). This suggests that surgical cytoreduction may not have the same value in the RPV-stratified subgroups. Across a range of thresholds for RPV-based stratification, we confirmed that the hazard ratio for tumor-free status (associating with either OS or PFS) was consistently higher in the RPV-high group (Supplementary Figure 8a). This analysis was limited by the small sample size and future validation in a larger prospective study is required. Nevertheless, it highlights the potential application of RPV in the surgical decision setting.

Although RPV was found associated with suboptimal resection, it did not accurately predict the resection outcome on its own, perhaps due to the influence of surgical skill on this variable (Supplementary Figure 9).

Construction of eRPV, an expression profile-derived RPV surrogate

Since the derived RPV correlated with the expression level of 82 genes (r< -0.4 or r>0.4, Spearman correlation), we asked whether expression profiles could be used as a surrogate marker of RPV to test its prognostic power and associated biology in more independent datasets. We applied feature selection and linear regression between gene expression profile and RPV in the TCGA dataset using LASSO, and discovered a gene expression signature (vector) that strongly correlated with RPV (Supplementary Figure 10a-c), which we named eRPV. eRPV showed comparable prognostic potential as RPV in the TCGA dataset (n= 69; HR= 11.5, 95% CI (0.923 – 143), p= 0.0578; Cox regression). The borderline association perhaps suggests that eRPV captures similar prognostic characteristics though not as comprehensive as RPV, likely due to incomplete reflection of total stroma content or structure. As we expected, eRPV statistically interacted with RPV in a multivariable Cox regression model whereas only the latter retained prognostic power (eRPV: p= 0.615, RPV: HR=6.33, 95% CI (1.51-26.5), p= 0.0115).

Taking advantage of the publicly available gene expression datasets, we demonstrated that eRPV was an independent prognostic factor in two additional patient cohorts (TCGA cohort devoid of publicly available CT scans: n=448, HR= 2.19, 95% CI (1.23 - 4.25), p= 0.0208; Tothill: n= 228, HR= 7.94, 95% CI (2.02 - 31.3), p= 0.00303; adjusted for stage, grade, residual disease, age and neo-adjuvant chemotherapy; Supplementary Figure 10d-10e). We obtained consistent results using gene expression profiles from two microarray platforms (Supplementary Figure 10f). eRPV developed from the RNA-sequencing dataset showed a consistent trend with two other platforms though not statistically significant, possibly due to the smaller sample size.

We further investigated the prognostic value of eRPV in five additional cancer types from the TCGA study (glioblastoma, lung squamous carcinoma, kidney clear cell carcinoma, breast invasive carcinoma and colorectal adenocarcinoma; Supplementary Figure 10f). We found that higher eRPV was significantly associated with poor overall survival in glioblastoma and showed a trend towards poor survival in lung squamous carcinoma, suggesting that these two cancer types may share similar prognostic characteristics as EOC, and that RPV may be transferrable to these cancer types.

Collectively, the gene-expression derived surrogate of RPV, eRPV, showed prognostic potential in two additional large independent cohorts which consolidates RPV as a prognostic marker in EOC. The prognostic value of RPV was at least partly recapitulated by eRPV, reiterating a robust biological basis for RPV.

Stromal cell content is associated with poor overall survival

Previous studies have reported a strong link between high stromal component and poor prognosis in EOC [3-6], therefore we validated the association between stromal content with overall survival using the TCGA dataset (Supplementary Figure 7f). We found that a higher percentage of stromal cells in

the tumor specimens assessed was significantly associated with poor overall survival (p= 0.0101, log-rank test). This association was further confirmed in the continuous Cox regression analysis adjusted for stage and post-operative residual disease (HR= 1.14, 95% CI (1.01-1.29), p= 0.041, Cox regression).

Association between RPV and molecular subtypes or BRCA mutations

Previous studies identified four consistent molecular subtypes of HGSOC, namely C1 (Mesenchymal), C2 (Immunoreactive), C4 (Differentiated) and C5 (Proliferative) [6, 7]. As the subtype-profile was prognostic for HGSOC (Supplementary Figure 11), we considered whether they would associate with RPV. We found that RPV had only a borderline association with the C1 (Mesenchymal) subtype in the TCGA cohort and not in the HH cohort. This finding was surprising as the C1 subtype is reported to be linked to stromal component. This may be explained by the distinct features of stroma measured by RPV and the C1 subtype: the former, derived from CT images, likely measures the stromal structure whereas the latter likely measures stromal activation. Interestingly, eRPV was strongly associated with the C1 subtype in both TCGA and Tothill dataset, conceivably due to eRPV being a gene expression signature as is the C1 subtype (Supplementary Figure 7i).

HGSOC is genetically driven by *TP53* mutation, defects in homologous recombination including *BRCA1/2* mutation and extensive CNA. We asked whether there were any upstream genetic driver events that could regulate RPV. We analysed the association between RPV and *BRCA1/2* mutations or CNA. Although no associations between RPV and germline *BRCA1/2* mutations or between RPV and CNA profiles were observed after adjusting for multiple testing, we observed a borderline association between eRPV and somatic *BRCA1/2* mutations (Supplementary Figure 7j). However, RPV showed a negative trend with total mutation burden and total CNA burden within the TCGA dataset (Supplementary Figure 7k-7l).

Association between RPV and *CCNE1* copy number status

Previous reports suggest that *CCNE1* gene amplification is associated with primary chemotherapy response and progression free survival in HGSOC [7-9], thus the question was posed whether RPV correlated with *CCNE1* amplification. Copy number data from the TCGA cohort and the HH cohort confirmed the previous association between *CCNE1* and PFS (TCGA: n= 426, HR= 1.33, 95%CI (1.00-1.77), p= 0.0472; HH: n= 114, HR= 1.61, 95%CI (0.914-2.82), p= 0.0994, Cox regression adjusted with stage and residual disease). However, we did not observe any association between RPV and *CCNE1* amplification in the TCGA or the HH cohort (TCGA: *r*= -0.0916, p= 0.472; HH: *r*= -0.0465, p= 0.663; Spearman correlation). These results suggest that the association between RPV and primary chemotherapy resistance was unlikely to be due to *CCNE1* gene amplification. It is possible that enhanced extracellular matrix deposition, reflected in RPV, may be a more relevant determinant of primary chemo-resistance in HGSOC in this context [8]. Thus, RPV could be potentially used in combination with *CCNE1* amplification to predict response to chemotherapy and select patients for alternative therapies.

Supplementary References

1. Micci, F. et al. Tumor spreading to the contralateral ovary in bilateral ovarian carcinoma is a late event in clonal evolution. *Journal of oncology* **2010**, 646340 (2010).
2. Vargas, H.A. et al. Radiogenomics of High-Grade Serous Ovarian Cancer: Multireader Multi-Institutional Study from the Cancer Genome Atlas Ovarian Cancer Imaging Research Group. *Radiology* **285**, 482-492 (2017).
3. Zhang, S.Z. et al. Stroma-associated master regulators of molecular subtypes predict patient prognosis in ovarian cancer. *Sci Rep-Uk* **5** (2015).
4. Chen, P. et al. Identification of Prognostic Groups in High-Grade Serous Ovarian Cancer Treated with Platinum-Taxane Chemotherapy. *Cancer research* **75**, 2987-2998 (2015).
5. Busuttil, R.A. et al. A signature predicting poor prognosis in gastric and ovarian cancer represents a coordinated macrophage and stromal response. *Clinical cancer research : an official journal of the American Association for Cancer Research* **20**, 2761-2772 (2014).
6. Tothill, R.W. et al. Novel molecular subtypes of serous and endometrioid ovarian cancer linked to clinical outcome. *Clinical Cancer Research* **14**, 5198-5208 (2008).
7. Cancer Genome Atlas Research, N. Integrated genomic analyses of ovarian carcinoma. *Nature* **474**, 609-615 (2011).
8. Etemadmoghadam, D. et al. Integrated genome-wide DNA copy number and expression analysis identifies distinct mechanisms of primary chemoresistance in ovarian carcinomas. *Clinical cancer research : an official journal of the American Association for Cancer Research* **15**, 1417-1427 (2009).
9. Patch, A.M. et al. Whole-genome characterization of chemoresistant ovarian cancer. *Nature* **521**, 489-494 (2015).
10. Phelps, D.L. et al. Methylation of MYLK3 gene promoter region: a biomarker to stratify surgical care in ovarian cancer in a multicentre study. *Br J Cancer* **116**, 1287-1293 (2017).
