# Supplementary material for: A mathematical-descriptor of tumor-mesoscopic-structure from computed-tomography images annotates prognostic- and molecular-phenotypes of epithelial ovarian cancer
Source: Nat Commun. 2019 Feb 15;10:764. doi: 10.1038/s41467-019-08718-9 (PMC6377605; doi:10.1038/s41467-019-08718-9)
Supplement: Supplementary file 8 — Reporting Summary [file 41467_2019_8718_MOESM8_ESM.pdf]

nature research

Corresponding author(s): Prof Eric Aboagye

Last updated by author(s): 2018/12/14

Reporting Summary

Nature Research wishes to improve the reproducibility of the work that we publish. This form provides structure for consistency and transparency in reporting. For further information on Nature Research policies, see Authors & Referees and the Editorial Policy Checklist.

Statistics

For all statistical analyses, confirm that the following items are present in the figure legend, table legend, main text, or Methods section.

n/a	Confirmed	
☒	☐	The exact sample size (*n*) for each experimental group/condition, given as a discrete number and unit of measurement
☐	☒	A statement on whether measurements were taken from distinct samples or whether the same sample was measured repeatedly
☐	☒	The statistical test(s) used AND whether they are one- or two-sided<br>*Only common tests should be described solely by name; describe more complex techniques in the Methods section.*
☐	☒	A description of all covariates tested
☐	☒	A description of any assumptions or corrections, such as tests of normality and adjustment for multiple comparisons
☐	☒	A full description of the statistical parameters including central tendency (e.g. means) or other basic estimates (e.g. regression coefficient) AND variation (e.g. standard deviation) or associated estimates of uncertainty (e.g. confidence intervals)
☐	☒	For null hypothesis testing, the test statistic (e.g. *F*, *t*, *r*) with confidence intervals, effect sizes, degrees of freedom and *P* value noted<br>*Give P values as exact values whenever suitable.*
☒	☐	For Bayesian analysis, information on the choice of priors and Markov chain Monte Carlo settings
☒	☐	For hierarchical and complex designs, identification of the appropriate level for tests and full reporting of outcomes
☐	☒	Estimates of effect sizes (e.g. Cohen's *d*, Pearson's *r*), indicating how they were calculated

Our web collection on statistics for biologists contains articles on many of the points above.

Software and code

Policy information about availability of computer code

Data collection	A customized software, TexLab v2.0, was used to generate radiomic data in this study.
Data analysis	R 3.3.1 was used to analyze data in this study.

For manuscripts utilizing custom algorithms or software that are central to the research but not yet described in published literature, software must be made available to editors/reviewers. We strongly encourage code deposition in a community repository (e.g. GitHub). See the Nature Research guidelines for submitting code & software for further information.

Data

Policy information about availability of data

All manuscripts must include a data availability statement. This statement should provide the following information, where applicable:

- Accession codes, unique identifiers, or web links for publicly available datasets
- A list of figures that have associated raw data
- A description of any restrictions on data availability

The radiomics, clinical and proteomics data that support the key findings of this study are available in Mendeley Data with the identifier doi:10.17632/4c5znk5m8t.1. The gene expression, copy number alteration and RPPA data from the TCGA project55 were downloaded from UCSC cancer browser [https://genome-cancer.ucsc.edu/]. The gene expression microarray data from the Tothill dataset and laser capture microdissected ovarian tumour tissue were downloaded from the NCBI Gene Expression Omnibus with accession number GSE989114 and GSE4059556. The CT scan data from the TCGA ovarian cancer project were downloaded from the Cancer Imaging Archive45 [http://www.cancerimagingarchive.net/].

October 2018

Field-specific reporting

Please select the one below that is the best fit for your research. If you are not sure, read the appropriate sections before making your selection.

☒ Life sciences ☐ Behavioural & social sciences ☐ Ecological, evolutionary & environmental sciences

For a reference copy of the document with all sections, see nature.com/documents/nr-reporting-summary-flat.pdf

Life sciences study design

All studies must disclose on these points even when the disclosure is negative.

Sample size	The sample sizes of the two validation datasets were determined based on the effect size and confidence interval from the discovery dataset. Overall, 73 cases were needed after accepting the alpha of 0.05 and beta of 0.25. HR of 2.78, 31.6% cases in the high-risk group, median survival of 5 years in the low-risk group and median follow-up as 5 years. No statistical method was used to pre-determine the sample size of the molecular signatures association (gene expression, RPPA, targeted RNA-seq or copy number alterations). These data were collected based on the availability of tissue samples with appropriate tumor cellularity.
Data exclusions	No data were excluded during RPV development. Cases with poor tumor cellularity (<30%) were excluded in the analysis. The samples with poor tumor cellularity are often less representative of the corresponding tumor from previous experience, and they have distinct molecular characteristics from the rest of the samples. This exclusion criteria was pre-established.
Replication	The reproducibility of the prognostic power of RPV was validated in two additional datasets, including a multi-center cohort. The replication of RPV was successful in both validation datasets. The association between RPV and molecular signatures were replicated in at least two independent datasets to ensure reproducibility.
Randomization	The HH discovery and HH validation datasets were formed based on the radiomic data and treatment characteristics. The TCGA validation dataset was an independent dataset published previously. The clinical characteristics of the three datasets were compared in the manuscript. All the covariates that may cause bias (stage, age, residual disease and slice thickness) were controlled by being introduced as covariates in the multivariable Cox regression model.
Blinding	Investigators were blinded from group allocation during data collection.

Reporting for specific materials, systems and methods

We require information from authors about some types of materials, experimental systems and methods used in many studies. Here, indicate whether each material, system or method listed is relevant to your study. If you are not sure if a list item applies to your research, read the appropriate section before selecting a response.

Materials & experimental systems

n/a	Involved in the study	
☒	☐	Antibodies
☒	☐	Eukaryotic cell lines
☒	☐	Palaeontology
☒	☐	Animals and other organisms
☐	☒	Human research participants
☒	☐	Clinical data

Methods

n/a	Involved in the study	
☒	☐	ChIP-seq
☒	☐	Flow cytometry
☒	☐	MRI-based neuroimaging

Human research participants

Policy information about studies involving human research participants

Population characteristics	In the HH cohort, the median age is 62 (range 19-91); 53 cases with FIGO stage I-II and 223 cases with FIGO stage III-IV; 231 cases have serous histology and 58 cases have non-serous histology; 205 cases have optimal cytoreductive while 59 cases have sub-optimal cytoreductive surgery.
Recruitment	EOC patients included in the Hammersmith cohort were treated at the Hammersmith Hospital (HH), Imperial College London NHS Trust between June 2004 and November 2015. The patients were identified based on the availability of fresh frozen tumor tissue samples and pre-operative CT images.
Ethics oversight	Ethical approval for retrospective analysis of human data was obtained under the Hammersmith and Queen Charlotte's & Chelsea Research Ethics Committee approval 05/QO406/178.

Note that full information on the approval of the study protocol must also be provided in the manuscript.
