# Supplementary material for: A mathematical-descriptor of tumor-mesoscopic-structure from computed-tomography images annotates prognostic- and molecular-phenotypes of epithelial ovarian cancer
Source: Nat Commun. 2019 Feb 15;10:764. doi: 10.1038/s41467-019-08718-9 (PMC6377605; doi:10.1038/s41467-019-08718-9)
Supplement: Supplementary file 9 — Description of Additional Supplementary Files [file 41467_2019_8718_MOESM9_ESM.docx]

**Title:** Supplementary Data 1.
**Description:** Radiomic features assessed in this study and their corresponding class.

**Title:** Supplementary Data 2.
**Description:**  Results of Cox regression for each individual radiomic feature within the HH discovery dataset.

**Title:** Supplementary Data 3.
**Description:** Results of Gene Set Enrichment Analysis (GSEA) for RPVpositively correlated pathways.

**Title:** Supplementary Data 4.
**Description:** GSEA result for RPV-negatively correlated pathways. o Supplementary Data 5. Gene expression features from the Affymetrix U133 platform that contribute to eRPV
